# Instruments for cataract surgery: results from our survey

**Published:** 2011-12

**Authors:** Daksha Patel, Phil Hoare

**Affiliations:** MSc Course Director and Clinical Lecturer, International Centre for Eye Health (ICEH), London School of Hygiene and Tropical Medicine, Keppel Street, London WC1E 7HT, UK.; Procurement Manager, Sightsavers; IAPB Procurement Advisor and IAPB Standard List Administrator.

**Figure:**
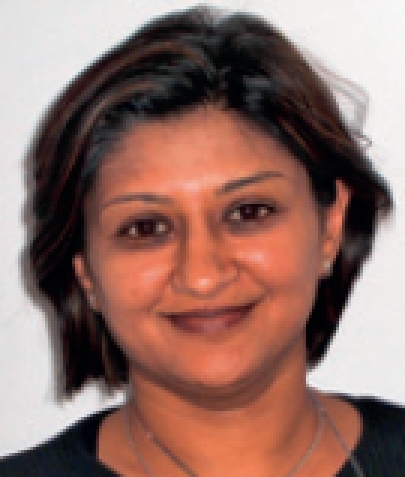
Daksha Patel

**Figure:**
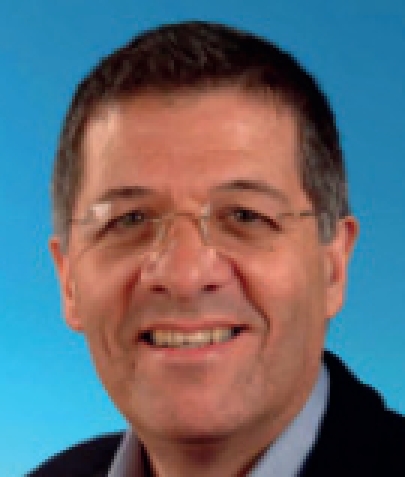
Phil Hoare

For any operation, including cataract surgery, the appropriate instruments must be available and in good working order. If instruments are not available, or are blunt, or do not function properly, it may be necessary to delay or postpone surgery. Using such instruments in an operation can result in a poor outcome, or even pose a risk to surgeons and their assistants.

The impact is therefore considerable, and can damage the reputation of the hospital in the community.

It may also mean that patients remain blind or have to travel further for treatment at an additional cost to them. They may even resort to using traditional methods, such as couching.

Making sure surgical instruments are in good working order requires the following:

- Purchasing high-quality instruments, as these are likely to be more robust and will last longer. The companies supplying them are also more likely to offer service warranties.
- Cleaning the instruments carefully after each operation, checking them to make sure they are still in good working order, packing them carefully, and sterilising them using appropriate methods.
- Checking that everyone working in the operating theatre and sterilisation areas knows how to handle instruments carefully. If sterilisation is centralised, those used to handling the large, robust instruments used in general surgery, orthopaedics, or obstetrics will have to be trained in handling ophthalmic instruments, which are small and delicate with fine points or very short blades.

Long-term maintenance of instruments requires an additional set of activities that must be carried out in a systematic, scheduled, and routine manner. These activites include:

- **Regular preventive maintenance**, which comprises inspection (preferably with magnification), cleaning, lubrication, and replacement at regular intervals
- **Record-keeping for maintenance**, so eye units can record which instruments are broken and must be repaired, when they broke, when they were repaired, and so on
- **Repair of broken or unusable instruments**, carried out on defective instruments or parts of instruments
- **Withdrawal and disposal**, a protocol for discarding instruments, trading old instruments for new, or updating and salvaging old instrument parts as spares
- **Spares planning**, which consists of anticipating which instruments will require replacement and keeping spares in stock to replace instruments without delay.

Long-term maintenance activities can also be supported by negotiating annual maintenance contracts with instrument suppliers, although not all suppliers will provide this service.

## Methods used

The survey discussed in this article was designed to find out:

- What instruments are used in different eye centres worldwide
- How they are cleaned and maintained
- Who is responsible for their care
- What effect poorly maintained, broken, or missing instruments had on the effectiveness of cataract services.

**Figure:**
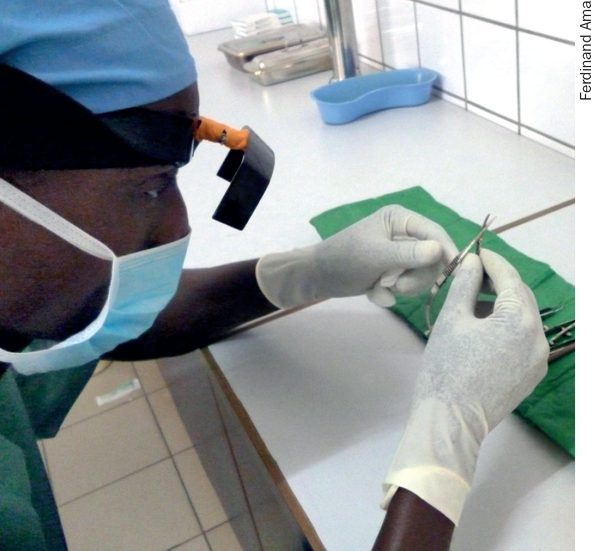
Regular preventive maintenance, such as cleaning and inspection, prolongs the life of instruments. IVORY COAST

The survey focused on the equipment needed for cataract surgery, but many of the principles apply to all eye surgery. We designed the survey using the Bristol Online Surveys (BOS) service. The IAPB Standard List (see page 30) provided a guide to the instruments needed for cataract surgery.

The survey was circulated to Sightsavers and CBM regional offices and to alumni of the International Centre for Eye Health (ICEH) Community Eye Health/Public Health for Eye Care MSc. It was also made available on the ICEH website. Only one entry per hospital was accepted and the data collection period was over three months, from 5 April to 3 July 2011. Data collected were cleaned and analysed using BOS and Microsoft Excel.

We asked eye units to provide information on the number of cataract operations performed in 2010 and on the number of ophthalmologists who worked there. Each unit was also asked to describe the roles and responsibilities of those handling surgical instruments and whether they had undergone any training.

Units were asked to report on a list of 41 instruments in four main groups: scissors, forceps, knives, and cannulae.

All the instruments required to perform a single operation are usually kept together as a complete ‘cataract set’.

Ideally, to ensure efficiency, there should be three complete cataract sets for every theatre bed in use. This means that, while one set is being used for surgery, another can be set up for the next patient and the third can be sterilised.

**Table 1.** Breakdown of high- and low-volume units by region

	**Africa**	**Southeast Asia**	**Other regions**	**Total**
Low-volume units (<=2,000 operations in 2010)	27	17	7	51
High-volume units (>2,000 operations in 2010)	5	26	3	34

We asked respondents whether any instruments were broken or unusable, the reasons why, and for how long they had not been usable.

We also asked how many units had incomplete cataract sets, and what the effect of this was on the services they were able to provide.

## Findings

A total of 85 eye units responded to the survey. Most were in Southeast Asia (43) and Africa (32). A total of 83 were from low- and middle-income countries, and two were from high-income countries. The responding units were funded by government, by non-governmental organisations (NGOs), or had combined funding from two or more sources.

The populations served by all the eye units ranged from under 0.5 million to over 3 million.

Sixty per cent of the eye units carried out small-incision cataract surgery (SICS) and 24% reported extracapsular cataract surgery (ECCE) as their main method. All had a high percentage of surgery with intraocular lens (IOL) implantation. Phacoemulsification was only performed in 5% of the eye units.

**Figure 1.**
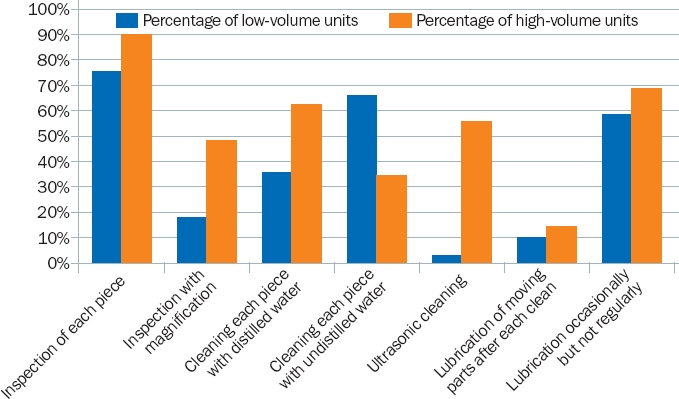
Comparing day-to-day instrument care in low- and high-volume units

During analysis, we divided the eye units into two broad groups, based on the number of operations performed in a year. Those performing over 2,000 operations were classed as ‘high-volume units’ and those performing fewer than 2,000 operations in 2010 were classed as ‘low-volume units’.

There were 51 low-volume units, most of which were from Africa, and 34 highvolume units, most of which were from Southeast Asia (see Table 1).

High-volume units were mainly funded from combined sources. Across all regions, 80% of those funded by government were low-volume units.

### Procurement

The vast majority of the responding eye units (89%) reported having a person in charge of procurement who followed a specific protocol.

### Instruments used

When the units' preferences were compared across the regions, it was clear that there was no one type of instrument that all units preferred.

However, capsulorrhexis forceps were used more commonly in the responding eye units in Southeast Asia (81%) than in Africa (51%).

### Care of instruments

The day-to-day care of instruments varied across the responding eye units (Figure 1).

Whereas 62% of the high-volume units reported using distilled water to clean instruments, this was the case in only 35% of low-volume units. These units still cleaned their instruments, but using water that had not been distilled.

Although 80% of all responding eye units regularly inspected their instruments (75% of low-volume and 90% of high-volume units), only 29% inspected instruments using magnification (18% low-volume and 48% high-volume).

A total of 63% of eye units lubricated their instruments occasionally, and only 11% reported lubricating instruments after each clean.

### Long-term planning and maintenance

**Figure 2.**
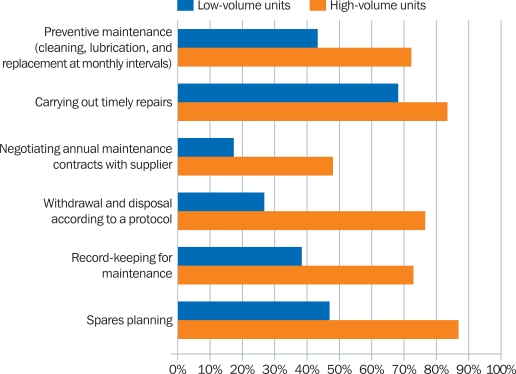
Comparing long-term planning and maintenance activities in low- and high-volume eye units

Overall, a greater proportion of the high-volume units who responded undertook long-term planning and maintenance activities (Figure 2).

In the questionnaire, these activities were described as ‘routine, periodic, pre-scheduled examination, repair, and replacement processes’:

- Preventive maintenance (cleaning, lubricating, and replacing broken parts at monthly intervals)
- Carrying out timely repairs
- Negotiating annual maintenance contracts with suppliers
- Withdrawing and disposing of instruments according to a set protocol
- Record-keeping for maintenance
- Spares planning.

### Who was in charge of instrument care?

Most eye units surveyed (81%) had someone in charge of instruments and consumables (70% in low-volume units compared to 97% in high-volume units).

In both settings, only half of those responsible for the maintenance and care of instruments had received any specific training for this role. Those who did not have any training either followed a protocol or worked under the supervision of an ophthalmologist.

Among the ten teaching hospitals who responded only half had a dedicated person in charge of instruments who had also received training for the role.

### Non-functioning instruments

**Figure 3.**
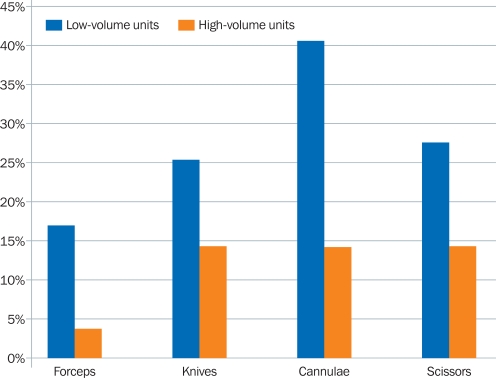
Percentage of eye units with non-functioning instruments, by surgical output

Compared to the high-volume units, considerably more of the low-volume units reported having non-functioning or unusable instruments (Figure 3).

In some low-volume units, five or more of the same kind of instrument did not work.

**Figure 4.**
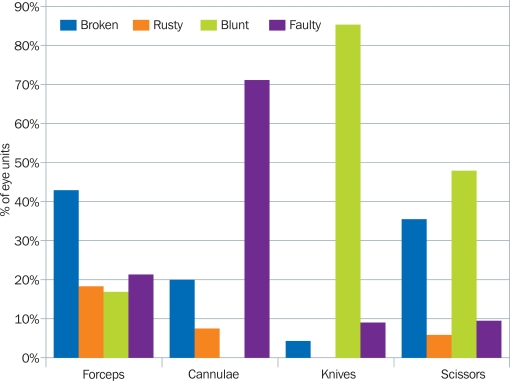
Main reasons that instruments were non-functioning, by group of instrument

The reasons given for instruments not working are summarised in Figure 4. Of those eye units who reported unusable forceps, the majority gave the reason as ‘broken’.

For cannulae, the most frequent reason was ‘faulty’; for knives, ‘blunt’. For scissors, both ‘blunt’ and ‘broken’ were reported very often.

Preventable causes (breakages due to poor handling, rust, being blunt) accounted for 73% of the unusable instruments.

In total, 40% of the eye units reported having instruments that had remained unusable for more than one year.

## Impact of non-functioning instruments

Where repair was a challenge or re-ordering slow, eye units had to delay surgery, ‘make do’ without these instruments, or use disposable options instead.

Almost half of the units from Asia and Africa (48%) had at least one incomplete cataract set. Overall, more low-volume than high-volume units reported incomplete sets (59% vs 28%). Overall, of the eye units based in Africa, 75% reported incomplete cataract sets, compared with just 21% of those in Asia.

**Figure 5.**
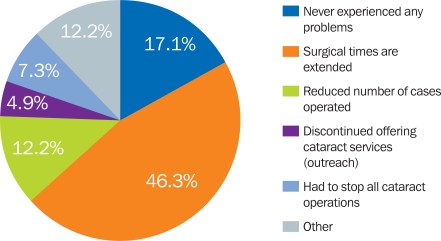
Impact of incomplete cataract sets

The impact of having incomplete sets was considerable (Figure 5): 46% of the responding eye units had to extend surgical times as they had to wait longer between operations for cataract sets to be cleaned and sterilised; 12% had reduced the number of cataract operations they performed, and 7.3% had discontinued outreach services.

Overall, 69% of eye units without a person responsible for instrument care had incomplete cataract sets, compared to 41% of units with a person responsible.

## Conclusions

Ultrasound cleaning is expensive, which may explain why so few of the responding low-volume eye units used it.

A greater proportion of the responding high-volume units conducted long-term maintenance activities, giving the impression that eye units doing high-volume surgery have a proactive and anticipatory approach to maintaining their instruments.

The fact that only half of the teaching hospitals who responded had a trained person in charge could be cause for concern.

This raises questions about the teaching hospitals' ability to demonstrate the appropriate quality standards for instrument care to surgeons and ophthalmic nurses during their training.

Good repair and maintenance regimes and protocols can minimise preventable causes such as rust, breakage due to poor handling (pages 36–37), and being blunt (see page 44).

Instruments that had remained unusable for a long time should have been replaced, repaired, or discarded. This can be addressed by putting in place both an instrument inventory where problems with instruments can be noted, and protocols to repair or withdraw these instruments.

Incomplete cataract sets affect surgical efficiency, as time is spent waiting to sterilise the available instruments between patients, or the unit is forced to reduce the overall number of operations done per surgical session.

In 5% of the responding eye units, outreach services were discontinued because of lack of instruments. This would have far-reaching consequences for people in remote settings.

**Figure:**
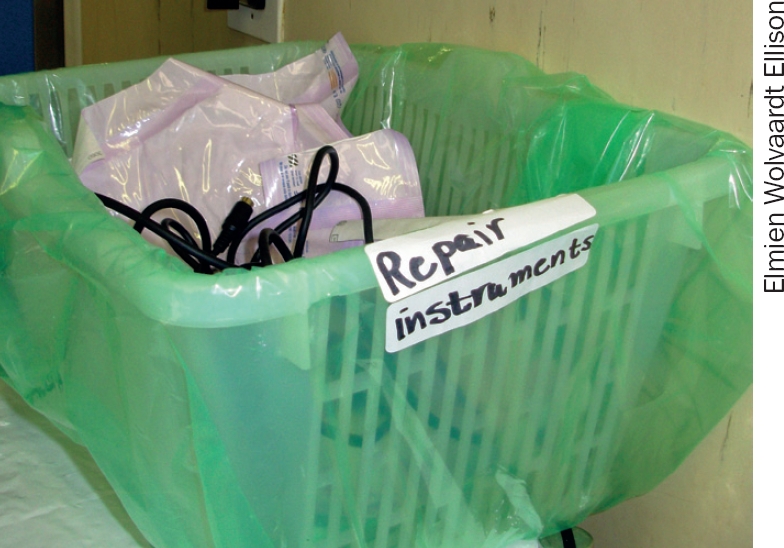
A special bin for non-functioning instruments. SOUTH AFRICA

The survey responses suggest a link between having someone in charge of instruments and having fewer incomplete cataract sets, which is better for output and efficiency. Compared to low-volume units, a greater proportion of the highvolume units had such a person, which would boost their output even further.

## Recommendations

- Assign responsibility to one person to manage the daily care and long-term maintenance of instruments. This can be on a part-time basis.
- The routine care of instruments after each operation should consist of cleaning and lubrication to prevent rust and prolong the life of the instrument (see pages 36–37).
- After washing instruments to remove debris, you must ideally rinse them with distillied (pH neutral) water. This reduces the risk of corrosion and chemical damage.
- Regular inspection, particularly with magnification, is important to detect instruments in need of urgent repair or replacement. These observations must be recorded and acted upon in a timely manner.
- Keeping records of all breakages and of instruments needing repair would make it possible to actively manage procurement and repair activities. A simple idea is to create a bin where staff can place non-functioning instruments as soon as they are noticed. These can then be recorded and dealt with in a systematic way.
- Timely procurement (page 38) is important to ensure that instruments are replaced as needed. If instrument replacement is planned and anticipated based on a repair log, then delays will be minimised.
